# The effects of co-exposure to methyl paraben and dibutyl phthalate on cell line derived from human skin

**DOI:** 10.1007/s43188-022-00151-3

**Published:** 2022-08-31

**Authors:** Katarzyna Miranowicz-Dzierżawska, Lidia Zapór, Jolanta Skowroń, Luiza Chojnacka-Puchta, Dorota Sawicka

**Affiliations:** Central Institute for Labour Protection-National Research Institute, Czerniakowska 16, 00-701 Warsaw, Poland

**Keywords:** Co-exposure, Interactions, Methylparaben, Dibutyl phthalate, Skin cells

## Abstract

Data on the cumulative effects of chemical substances are necessary for the proper risk assessment, but their availability is still insufficient. The aim of the study was to evaluate the cytotoxic effect of methyl paraben (MePB) and dibutyl phthalate (DBP) on the cells of the skin line (A431) and to compare the cytotoxic effects of the tested substances after single application to A431 cells with the effects of an equimolar/equitoxic (1:1) binary mixture of these compounds as well as their mixtures in ratio 1:3: and 3:1. On the basis of the obtained results, it was found that there were interactions between the tested compounds in terms of cytotoxic effect on A431, assessed on the basis of metabolic activity of cells (MTT test) and integrity of their cell membranes (NRU test). The obtained values of synergy coefficients (SI) and isobolographic analysis indicate that between the tested chemicals in a two-component equimolar mixture (1:1) there is a synergism of action, which, at a high DBP content in the mixture (> 50%) turned into antagonism. Observations using a holotomographic microscope show morphological changes in A431 cells after exposure to both DBP and MePB separately and binary mixtures of these compounds, compared to untreated cells. The observed changes in cell morphology seem to be more pronounced when the cells are exposed to the binary mixtures of DBP and MePB than when exposed to these substances individually, which may confirm the synergy of cytotoxic activity between them (this phenomenon was observed for the higher of the tested concentrations in all tested proportions). It is important to consider such effects when considering the effects of cumulative exposure in the risk assessment in order not to underestimate the risk of adverse effects associated with exposure to chemical mixtures.

## Introduction

The combined action of two or more chemicals within any system is called an interaction. The theoretical potential for chemical interactions is beyond predictions, hence the studies on combined toxic effects seem still important and a difficult task of the current toxicology of practical importance. Interactions involve assessing possible modifications of the toxic effect of one substance by another, and the mechanisms responsible for these modifications. Toxicological interactions between chemicals can be toxicokinetic in nature, involving biotransformation, intra-systemic distribution and excretion, or toxicodynamic in terms of additivity, synergism and potentiation, which significantly affects health risk assessment. Additionally, different directions of interaction (synergism or antagonism) may be observed for mixtures of the same compounds, depending on the concentrations of the compounds in the mixture. Therefore, the risk assessment related to the effects of chemical compounds, based on data for individual substances, may lead to an underestimation of the risk, so the results of their effects should always be estimated on the basis of combined exposure studies.

The problem of cumulative effects in exposure to reprotoxic substances, including endocrine disrupting chemicals (EDCs) has long been considered important, but the data on their possible interaction in cases of combined exposure are frequently unavailable. The observations of Yang [1] which stated that more than 95% of toxicological research resources are devoted to the effects of single chemicals with almost complete neglect of testing mixtures are valid to this very day. This insight also applies to EDCs. More importantly, modification of the result of combined exposure to endocrine disrupting compounds can be expected even at doses well below the NOAEL (no observable adverse effect level) [2] and the results of research on possible interactions between reprotoxic substances or endocrine disruptors available in the literature indicate an ambiguous direction of changes in their action in mixtures.

It should be noted that the inclusion of the combined action of chemicals in the risk assessment has not been comprehensively regulated in European Union law so far. The combined effects of chemicals are considered for complex chemical products placed on the market (chemical mixtures), while the combined effects are not considered for simultaneous exposure to multiple xenobiotics present in the environment (including the work environment). The issue related to the assessment of the simultaneous effects of chemicals containing two or more substances on human health and the environment were addressed in the conclusions of the EU Environment Council of 22 December 2009 (*Council conclusions on combination effects of chemicals 2988th Environment Council meeting Brussels, 22 December 2009*). This document is not legally binding, but it seems to indicate the direction of actions that will be taken in relation to the issue at the European Union level. The Conclusions of the EU Environment Council include, inter alia, a proposal to consider the impacts from the combined action of a chemical when carrying out a chemical risk assessment. It is also suggested that further research is needed to obtain more information on this topic.

In the light of the current literature on this subject, which classifies both phthalates [3–5] and parabens [5, 6] as a group of endocrine disrupting chemicals, it has become important to carefully assess their combined effects as well, especially since the disruption of endocrine homeostasis can lead to multidirectional implications causing impairment of the body's efficiency and functions, and combined exposure to the above compounds is common [7–10]. Parabens belong to the portfolio of the most popular and most commonly used preservatives in cosmetics and/or drugs [11, 12], such as creams, soaps, perfumes and deodorants, hair care products, shaving and depilatory products, makeup, nails and agents with UV filters. Phthalates are used in the production of a very wide range of products that are intended for both professional users and the general population. These include cosmetics (hair or nail polishes, perfumes, deodorants, shampoos), pharmaceuticals (time-release capsule casings), medical devices (dialysis machines, blood containers), clothes (rainwear), plastics, food packaging, cleaning agents, printing inks or building materials, paints, varnishes and adhesives. It should be noted that the risk is further increased by the fact that during the production, transport or their improper disposal, phthalates penetrate into the soil, water and air, and the presence of phthalates has been found, for example, in many food products, which is related to the migration of these compounds from packaging to food stored in them [13].

Combined exposure may occur through ingestion, inhalation and dermal exposure. Both phthalates and parabens can permeate the skin. According to the definitions of Marzulli et al. [14] parabens would be classified as “moderate” penetrants. Seo et al. [15] observed that permeability coefficient (Kp) value of methylparaben (MePB) showed the highest value (comparing with propylparaben and butylparaben). The absorption rate of methylparaben in the pig-ear skin was higher from emulsions than from hydrogels, from enhancer-containing vehicles than from enhancer-free vehicles, and when skin was damaged [16]. Also dibutyl phthalate (DBP) could penetrate across skin (because it is metabolized by esterases in the skin) and can induce apoptosis of keratinocytes and fibroblasts via caspase-3 activation. [17]. Median exposure level to DBP in cosmetics by dermal absorption were estimated [18] to be 0.103 µg/kg body weight/d. Sugino et al. [19] noticed that when DBP was applied to skin of hairless rats and humans, only monobutyl phthalate appeared through the skin (the permeability of the skin was higher than after the application of the monoester directly to the skin). The skin has become impermeable to the metabolite following dermal exposure to dibutyl ester due to the inhibition of skin esterases. Intriguingly, removal of the stratum corneum from the skin did not change the skin permeation behavior. Taking into consideration that the mapped on a global scale cumulative phthalate exposure index (PEI) based on the phthalate pollution index (PPI) shows that phthalates are heterogeneously distributed globally, and about 30% of total environmental phthalates are ultimately exposed to the average human being [20] and Zao’s et al. [21] conclusion that currently, most studies on indoor phthalate exposure focus on inhalation and ingestion, and since the dermal exposure studies have received less attention, their exposure pathway has been underestimated. To date, information on this area are limited; therefore, further studies are still needed.

The aim of our study was to evaluate the cytotoxic effect of methyl paraben (MePB) and dibutyl phthalate (DBP) on the cells of the skin line (A431) and to compare the cytotoxic effects of the tested substances with the effects of the binary mixtures of these compounds. Providing the knowledge on the risks resulting from the combined action of chemicals is supposed to lead up towards raising awareness and contribution to the improvement of working conditions and reduction of the number of people exposed to dangerous mixtures (professional users and all consumers).

## Materials and methods

### Materials

The cytotoxicity of two substances with a proven harmful effect on reproduction and/or endocrine-disrupting metabolism was assessed—individually and in two-component mixtures in different molar proportions. The test substances methyl 4-hydroxybenzoate (methyl paraben) (MePB) (CAS 99-76-3) and dibutyl phthalate (DBP) (CAS 84-74-2) were purchased from Merck Millipore and Sigma-Aldrich (Sigma Chemical Company, St. Louis, MO, USA).

### Cell culture and reagents

The studies were performed on human *epidermoid (skin) carcinoma cell line A431* (ATCC^®^ CRL-1555). Cell line was purchased from LGC Standards, which distributes ATCC (American Type Cultures Collection) cultures.

A431 (ATCC^®^ CRL-1555) cells were cultured in Dulbecco's Modified Eagle Medium (DMEM) containing 1% antibiotic–antimycotic: penicillin G sodium, streptomycin sulphate, amphotericin B and fungizone (Sigma-Aldrich) and supplemented with 10% of foetal bovine serum obtained from Gibco BRL (Life Technologies Ltd., Paisley, Scotland) in sterile tissue culture flasks (Nunc, Roskilde, Denmark). Cells were maintained in monolayer cultures at 37 °C in a humidified atmosphere (5% CO_2_) and to detach them from the culture flasks, 0.25% trypsin–EDTA (Gibco) was used. Cells were checked for *Mycoplasma sp.* infection using a Myco-Alert™ PLUS Mycoplasma Detection Kit (Lonza, Walkersville, Inc.).

Before starting the experiments, cell viability in the suspension was assessed using a trypan blue dye exclusion test [22]. Suspensions were dispensed into a glass Bürker chamber and viable (trypan blue negative) and dead (trypan blue positive) cells were counted. The evaluation was carried out using a light microscope with inverted optics (Nikon TMS-F, Japan), employing a 100-fold magnification. Cell suspensions in which the number of faulty cells did not exceed 5% were used for further investigation.

In order to increase the solubility of the test compounds, which would allow the cells to be exposed to higher concentrations of xenobiotics, DMSO was used as their solvent, after determination of its non-toxic concentration to the exposed cells.

Cell cultures were seeded at a density of 1 × 10^5^ viable cells/ml with 100 µl of medium in each well of the 96-well microplates (Nunc, USA) and cultured overnight to allow adherence and recovery from exposure to trypsin. Next, the medium was aspirated from the cells and a solution of the tested compound (100 µl) was added to each well of the microplate. Each sample was applied in nine replications. Control cells were incubated in a nutrient medium without xenobiotics. Microplates were placed in a CO_2_ incubator for 24 h, after which the assays were performed.

The exposure of A431 cells to mixtures containing the following concentrations were tested.

- 85% MePB and 15% DBP (stock solution contained 340 mM MePB and 60 mM DBP in 5 ml DMSO)
- 75% MePB and 25% DBP (3:1) (stock solution contained 300 mM MePB and 100 mM DBP in 5 ml DMSO)
- 50% MePB and 50% DBP (1:1) (stock solution contained 200 mM MePB and 200 mM DBP in 5 ml DMSO)
- 25% MePB and 75% DBP (1:3) (stock solution contained 100 mM MePB and 300 mM DBP in 5 ml DMSO)
- 15% MePB and 85% DBP (stock solution contained 60 mM MePB and 340 mM DBP in 5 ml DMSO).

### Cytotoxicity tests

The following methods were used to evaluate the toxic effects of the test compounds and their binary mixtures on cells in vitro.

- MTT tetrazole salt reduction test determining the metabolic activity of cells (MTT test);
- Neutral Red Uptake Assay to assess the integrity of cell membranes (Neutral Red Uptake Assay, NRU test) according to INVITTOX Protocol No 17 and No 64, respectively.
- Clonogenic assay determining the ability of cells to proliferation (CFEA test).

### MTT assay

The in vitro cytotoxicity of single substances and their binary mixtures was evaluated using MTT assay as described by Mossman [23]. This assay is based on the assessment of the cells metabolic activity expressed by the ability to absorb yellow MTT tetrazolium salt and reducing it by mitochondrial succinate dehydrogenase to an insoluble formazan compound.

In the first step, cells were seeded at a density of 10,000 cells/well in a 96-well culture plate (Nunc) and cultured overnight (37 °C, 5% CO_2_). Then non-attached cells were aspirated and the rest of the cells were treated with various concentrations of the test substance/mixture and after removing the medium, medium-containing MTT (0.5 mg/ml in Hank’s buffered saline) was added to each well (100 µl). After 3 h incubation, supernatants were removed and the purple-blue formazan product was dissolved in 100 µl of DMSO (Gibco). Absorption was measured using an ELISA microplate reader (at 570/620 nm), after shaking the plate for 3 min before the measurement. On the basis of the results, it was determined that the concentration of the test substance/mixture caused a decrease in metabolic activity of cells by 50% (IC_50_) compared to the control. Cytotoxicity assays were performed in three iterations. IC_50_ values for each tested compound/mixture were calculated by a computer program using interpolation curves (four logistics) KCjunior (version 5), BioTek Instruments, Inc.

### NRU assay

The principle of the NRU assay is based on the ability of live, intact cells to absorb the dye, neutral red (3-amino-7-dimethylamino-2-methyl phenazine hydrochloride) accumulated in the lysosomes. Similarly to the MTT test, cells were first seeded at a density of 10,000 cells/well in a 96-well culture plate (Nunc) and cultured overnight (37 °C, 5% CO_2_). Then, non-attached cells were aspirated and the rest of the cells were treated with various concentrations of the test substance medium-containing neutral red dye (100 µg/cm^3^) added to each well (100 µl). After 3 h incubation, the supernatants were removed and the cells were treated with 100 µl of solution containing 50% ethanol, 49% distillate water and 1% glacial acetic acid. The cell membranes were destroyed in order to release the dye. The concentration of the dye was determined spectrophotometrically using an ELISA microplate reader (at a wavelength of 540/450 nm). On the basis of the results, it was determined that the concentration of test substance inhibited the ability of cells to absorb the dye by 50% (IC_50_) compared to the control.

Based on the MTT and NRU tests for the tested equimolar (1:1) binary mixture, the synergy index (SI) was calculated [24], defined as:

$$SI = \frac{{E}_{A+B(obs.)}}{{E}_{A+B(calc.)}}$$

where, E_A+B(obs.)_ is the observed/real effect of the A + B mixture, E_A+B(calc.)_ is the calculated/expected effect of the A + B mixture assessed on the basis of the effects of individual substances.

The theoretical IC_50_ values for the two-component mixture were calculated as the sum of the products of the mean IC_50_ values determined for each compound separately and the percentage of the compound in the mixture.

When synergy is estimated on the basis of IC_50_ values that are inversely proportional to the toxic effect of the substance, a synergistic effect is found when SI < 1.

### CFEA test

Clonogenic assay was used to determine the ability of a cell to proliferate indefinitely, thereby retaining its reproductive ability to form a large colony or a clone. This cell is then said to be clonogenic [25]. Clonogenic assay, also known as colony-forming efficiency assay (CFEA), is based on the ability of a single cell to grow into a colony [26], where the colony is defined as to consist of at least 50 cells [27].

The clonogenic assay was conducted according to the procedure described by Franken et al. [27] and adapted from Kruszewski et al. [28]. Cells in the exponential growth phase were harvested and seeded in a Petri dish of 60 × 15 mm (21 cm^2^) (Iwaki Cell Biology, Japan) at a density of 500 cells/dish in 5 ml of medium-containing tested substance/mixture in appropriate concentrations. Each experiment was performed in three independent replicates. Cells were exposed to the tested substance/mixture for 7 days. After this period, the medium was removed, and the cells were washed with PBS. Then, the colonies were fixed with ethanol (Sigma-Aldrich), stained with Giemza solution (0.4%, Sigma-Aldrich) and counted using a stereomicroscope (IUL, Spain). The plating efficiency (PE) and surviving fraction (SF) were calculated, as below:

$$\text{Plating efficiency }(\text{PE}) = \frac{\text{number of colonies counted }}{\text{number of cells plated}}$$

$$\text{Surviving fraction }\left(\text{SF}\right)=\frac{\text{number of colonies counted after exposure }}{\text{number of cells plated }\times \text{ plating efficiency}}$$

The SF for the control cells were understood to be equal to 1.

### Visualisation of morphological changes in exposed cells using HTM

Morphological changes in the cells caused by the test compounds individually and their mixtures were assessed by holotomographic microscopy (HTM). To visualise mitochondrial structures, the cells were incubated with MitoView (Biotium) dye at 100 nM, while nuclei were stained with DAPI reagent (1: 1000, Sigma). The cells were incubated with the above-mentioned dyes for 15 min at 37 °C. After staining the cell structures, observations of the cells were carried out under a holotomographic microscope.

### Apoptosis markers assessement

Assessment of caspase-3/7 activity was performed for 24 h using a IncuCyte^®^ S3 Live-Cell Analysis System as well as using a fluorescence image cytometer NucleoCounter^®^ NC-3000™ to determine caspase-3/7 enzymatic activity by FLICA (Fluorochrome-Labelled Inhibitors of Caspases).

The translocation of phosphatidylserine in the cells was also assessed using NC 3000 on the basis of binding the fluorescently labelled Annexin V protein.

### Caspase 3/7 assay using IncuCyte

A431 cells were seeded at a density of 5000 cells per well in 96-well plates (Nunc) and incubated overnight at 37 °C in a humidified atmosphere (5% CO_2_). Next, the cells were exposed to different concentrations of the tested substance/mixture for 24 h. Next, fresh medium containing 1 × IncuCyte Caspase-3/7 Apoptosis Assay Reagent (Sartorius) were added. Before starting the scanning, plates were incubated (37 °C, 5% CO_2_) for 15 min. Cells treated with staurosporine (0.1 nM, Sigma-Aldrich) were used as a positive control. At each time point, five images were taken per well in both brightfield and FITC channels. The images were analysed for the number of green objects (fluorescing cells) per well by the algorithm in the IncuCyte S3 Software (v2018B). Each experiment was performed in three independent replications.

### Evaluation of two apoptosis markers in cells exposed to the tested substances/mixtures using Nucleocounter NC 3000

The translocation of phosphatidylserine in cells was also assessed using Nucleocounter NC 3000 on the basis of binding the fluorescently labelled Annexin V protein.

Measuring the binding of Annexin V conjugated with fluorescein-FITC isotiocyanate allows the detection of conformational changes of the cell membrane and loss of asymmetry in membrane lipid distribution, which is associated with phosphatidylserine translocation (the main component of membrane phospholipids) to the outer membrane layer without impinging on its integrity (an early marker of apoptosis). In order to differentiate apoptotic cells from necrotic cells, DNA staining with propidium iodide (PI) was used, which penetrates the damaged membrane of dead cells but does not penetrate the intact membranes. The total cell population was also labelled using a Hoechst 33342 reagent for the detection of all cells.

Determination of the enzymatic activity of caspase-3/7 by the FLICA method is used to detect apoptosis based on the assessment of caspase activity in situ in cells. The assay involves the use of a carboxyfluorescein-labelled (FAM) four-amino acid (Asp-Glu-Val-Asp) substrate that is conjugated with a fluoromethyl ketone residue (FMK), which determines its penetration through the cell membrane into the cell interior and irreversible binding of the specific amino acid sequence to the active centre of the caspase. Any unbound inhibitor is washed away and as a result labelled, and only cells in which the caspase has been activated are detected using a fluorescence reader [29].

The w/v or v/v solutions of the tested substances were prepared immediately prior to exposure of the cells. The solutions of each xenobiotic were prepared in two concentrations corresponding to 1/4 and 1/10 of the average IC_50_ determined experimentally. The level of apoptosis was tested in at least three replications.

The cells were suspended in the culture medium at the concentration required for the assay, and then placed in a Petri dish—vol. 5 ml. After 24 h incubation (37 °C, 5% CO_2_, 95% humidity), during which the cells were stuck to the bottom of the dish, the medium was aspirated and a solution of the test substance of a certain concentration in the culture medium was added to each plate. The dishes were placed in a CO_2_ incubator for 24 h, after which tests were performed. Negative control were non-exposed cells, while positive control cells were exposed to 0.1 nM staurosporine (the concentration of the compound was chosen experimentally).

#### Annexin V test

After incubation with the test compound, the cells were harvested and their concentration determined, followed by centrifugation at 400×*g* for 5 min. The pellet was resuspended in 300 μl PBS and centrifuged again under the same conditions. Then 2 ÷ 4 × 10^5^ cells were suspended in 100 μl working buffer, in which the Annexin V binding occurred and 2 μl of the Annexin V-FITC complex was added (labelling was performed under conditions that took into account the photosensitivity of the complex). Cells were then labelled with 2 μl of Hoechst 33342 reagent at 500 μg/ml and incubated at 37 °C for 15 min. in the heating block. After incubation, the cells were centrifuged at 400×*g* for 5 min. After removal of the supernatant, the pellet was resuspended in 300 μl working buffer, mixed and centrifuged. The procedure was repeated twice. The obtained cell pellet was resuspended in 100 μl of working buffer, 2 μl of 500 μg/ml propidium iodide solution was added, immediately placed on NC-Slide A2™ and analysed using a NucleoCounter NC-3000 with fluorescence microscope.

#### Determination of caspase-3/7 activity

After the incubation with the test compound/mixture, the cells were harvested and brought to a concentration of 2 ÷ 5 × 10^6^ cells/ml. Then 5 μl of FLICA reagent (previously reconstructed by the addition of 50 μl DMSO) diluted in PBS in a ratio of 1: 5, was added to the cell suspension (93 μl). Then 2 μl of Hoechst 33342 reagent at a concentration of 500 μg/ml was added and incubated at 37 °C for 60 min. in the heating block. After incubation, the cells were washed twice with 400 μl washing buffer, followed by centrifugation at 400×*g* for 5 min. After the final rinse, the cell pellet was resuspended in 100 μl washing buffer, supplemented with propidium iodide (PI), so as to obtain a concentration of 10 μg/ml, immediately placed 30 μl in the NC-Slide A2™ slide and analysed using an image cytometer NucleoCounter NC-3000 with fluorescence microscope. Based on fluorescence measurements, the percentage of cells without apoptosis, early and late apoptotic and necrotic was calculated.

### Statistical analysis

In the statistical analysis, firstly, descriptive statistics as well as one-sample t-test and one-sample Wilcoxon test were used. Listed tests were applied to compare the results of MTT, NRU and CFEA with theoretical values of these parameters at different levels of exposure to specific substances (DBP and MePB), with a different composition of mixtures. Additionally, using one-way ANOVA, a comparison was made of the results for Annexin V and caspases for subgroups distinguished by the composition of mixtures and two control groups. Effect size was measured by the partial eta-square (${\eta }^{2}$). In the statistical analysis, the commonly significance level of α = 0.05 was applied. The calculations were performed in the IBM^®^ SPSS^®^ Statistics 25.0 statistical package.

## Results

### MTT and NRU tests results

**Fig. 1:**
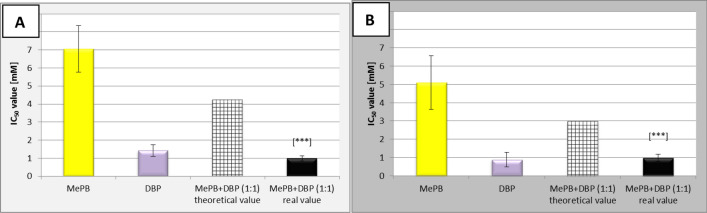
Comparison of experimentally determined IC_50_ values [mM] for an equimolar (1:1) mixture of methyl paraben (MePB) and dibutyl phthalate (DBP) with the theoretical values in MTT (**a**) and NRU (**b**) tests. The theoretical IC_50_ values for the two-component mixture were calculated as the sum of the products of the mean IC_50_ values determined for each compound separately and the percentage of the compound in the mixture. Data are presented as the mean ± SD, probability in one sample t-test: ***p < 0.001

By comparing the IC_50_ values obtained for individual substances after exposure to cells derived from human skin (A431), it was found that dibutyl phthalate (DBP) was more toxic for these cells, while methyl paraben (MePB) was less toxic (the mean IC_50_ values of DBP was established as 1.43 mM and 0.89 mM in MTT and NRU test, respectively; the mean IC_50_ values of MePB was established as 7.05 mM and 5.10 mM in MTT and NRU test, respectively). This finding was found both in the test assessing the metabolic activity of cells (Fig. 1a) and the integrity of cell membranes (Fig. 1b). The same pattern of cytotoxicity was observed in cells derived from human lung (A549) (data unpublished). Through the comparison of the IC_50_ values obtained after exposure of A431 cells to a binary equimolar (1:1) mixture of MePB and DBP with the theoretical values, calculated on the basis of the percentage content of the individual components in the mixture and their toxicity after each of them was administered individually to the cells of this line, it was found that the real value for the mixture was lower than the theoretical calculated value. This phenomenon was observed in both cytotoxicity studies (Fig. 1a, b).

Table 1 shows the SI values for the two-component equimolar mixture tested in the MTT and NRU tests. For the mixture of methyl paraben (MePB) with dibutyl phthalate (DBP) (1:1) in both tests performed, low values of the SI index were obtained, which indicates the synergistic effect of these compounds in the mixture.

**Table 1:** The value of synergy index (SI) calculated for equimolar mixture (1:1) of MePB and DBP in MTT and NRU tests

Mixture (1:1)	MTT test	NRU test
MePB + DBP	0.233	0.331

**Fig. 2:**
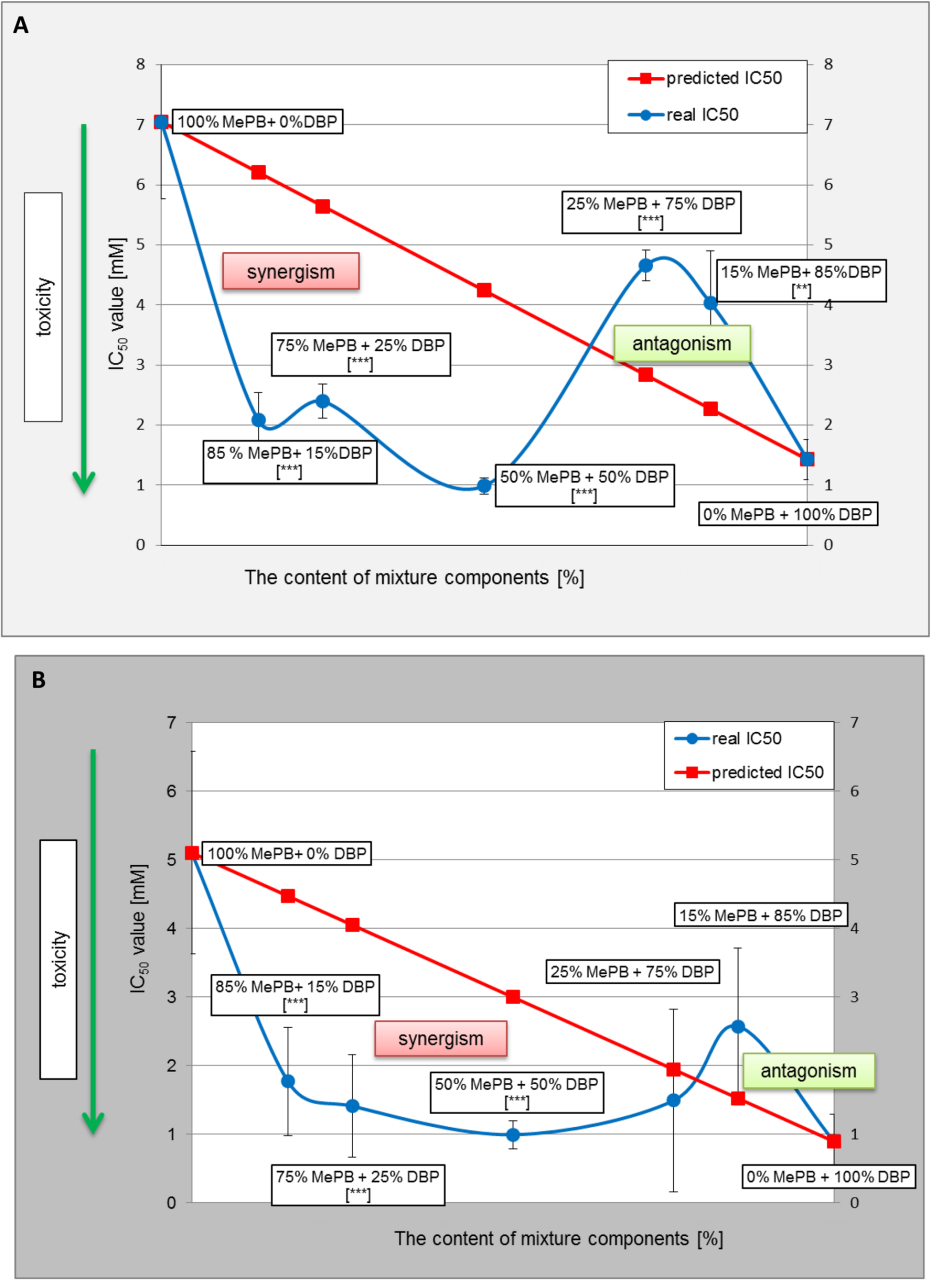
**a** Graphical interpretation of the combined effect of methyl paraben (MePB) and dibutyl phthalate (DBP) versus theoretical values as assessed by the MTT test. The isobole represents IC_50_ values determined in MTT test for an equimolar mixture of MePB and DBP and four mixtures in different ratios in comparison with theoretical values. The theoretical line of additivity is the straight line connecting the individual doses of each of the single agents that produce the effect alone. Direction of interactions between the components of the tested mixture was depended on the quantitative ratio of its components. In the case of the mixture with the dominant content of MePB (including the equimolar mixture), there was a synergistic effect, while with a higher content of DBP, the direction of the interaction changed to an antagonistic one. Data represent the mean ± SD, probability in one sample t-test: **p < 0.01, ***p < 0.001. **b** Graphical interpretation of the combined effect of methyl paraben (MePB) and dibutyl phthalate (DBP) versus theoretical values as assessed by the NRU test. Similarly as in (**a**) the isobole represents IC_50_ values determined in NRU test for an equimolar mixture of MePB and DBP and four mixtures in different ratios in comparison with theoretical values. The theoretical line of additivity is the straight line connecting the individual doses of each of the single agents that produce the effect alone. Data represent the mean ± SD, probability in one sample t-test: ***p < 0.001

The observation regarding the possibility of a synergistic effect between the components of the mixture of methyl paraben and dibutyl phthalate was verified on the basis of isobole images (graphical representation of interactions) plotted on the basis of the results of cytotoxicity determination for an equimolar mixture and four mixtures in different ratios (Fig. 2a, b). On the grounds of the graphical analysis of interpretation of the combined effect of methyl paraben (MePB) and dibutyl phthalate (DBP) in equimolar and non-equimolar mixtures based on the MTT test (Fig. 2a) in comparison with theoretical values, it was found that the direction of interactions between the components of the tested mixture depends on the quantitative ratio of its components. In the case of the mixture with the dominant content of methyl paraben (including the equimolar mixture), there is a synergistic effect, while with a higher content of dibutyl phthalate, the direction of the interaction changes to an antagonistic one. Due to the large standard deviation of the results of the assay, this was not found to be correct in the case of the cell membrane integrity (NRU) assay (Fig. 2b).

### CFEA test results

**Fig. 3:**
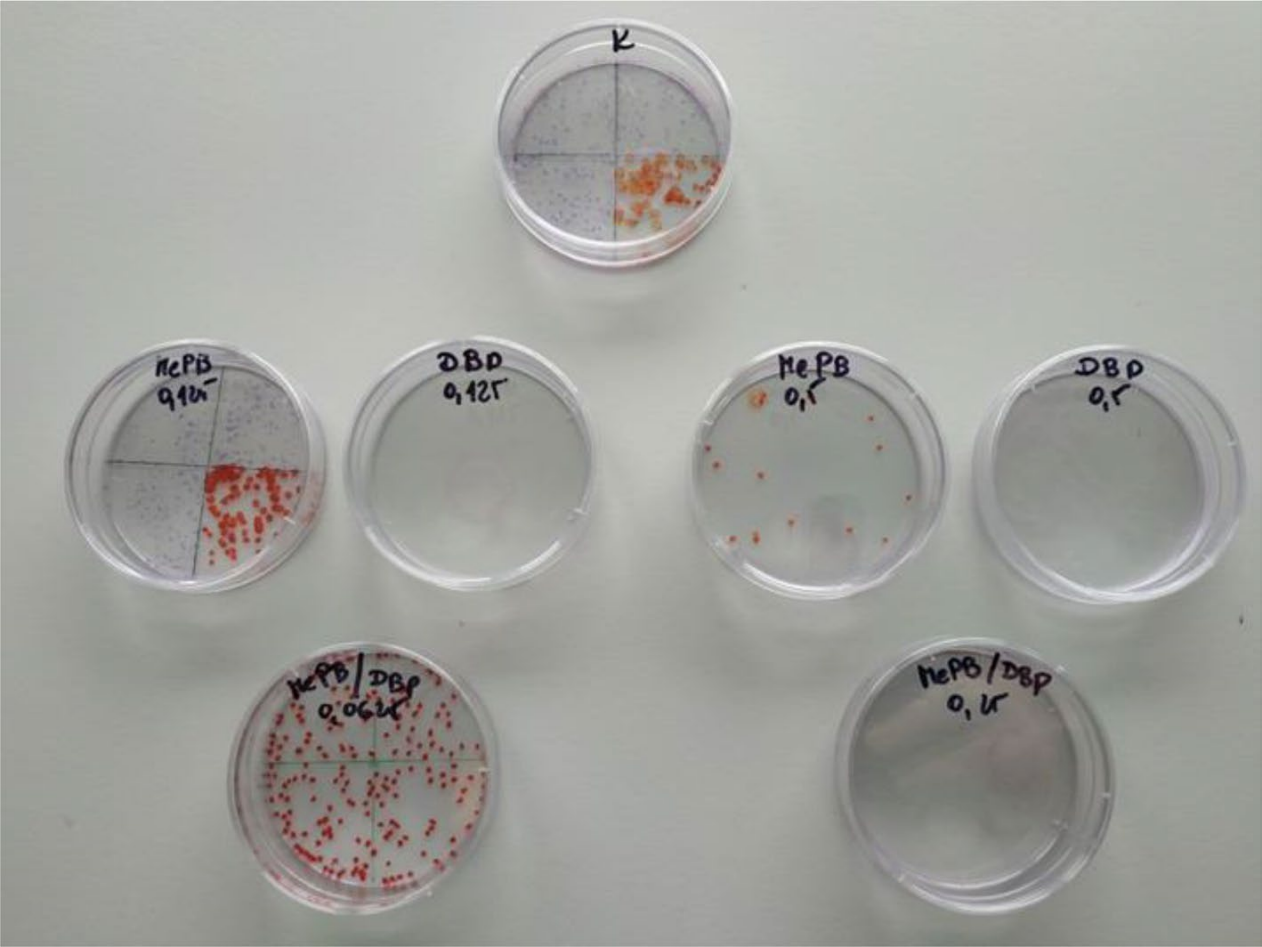
The pattern of CFEA test for A431 cells exposed to methyl paraben (MePB) and dibutyl phthalate (DBP) individually and in equitoxic mixtures of these compounds. Cells were exposed to the tested substance/mixture for 7 days, then colonies were fixed with ethanol (Sigma-Aldrich), stained with Giemza solution (0.4%, Sigma-Aldrich) and counted using a stereomicroscope (IUL, Spain). DBP applied individually to A431 cells, both at a concentration corresponding to 1/8 IC_50_ and at a concentration of ½ the value of the determined IC_50_ concentration, completely inhibited cell proliferation. MePB was slightly less inhibitory at a concentration corresponding to 1/2 IC_50_ value whereas at a concentration of 1/8 of its IC_50_ it almost did not inhibit proliferation

**Fig. 4:**
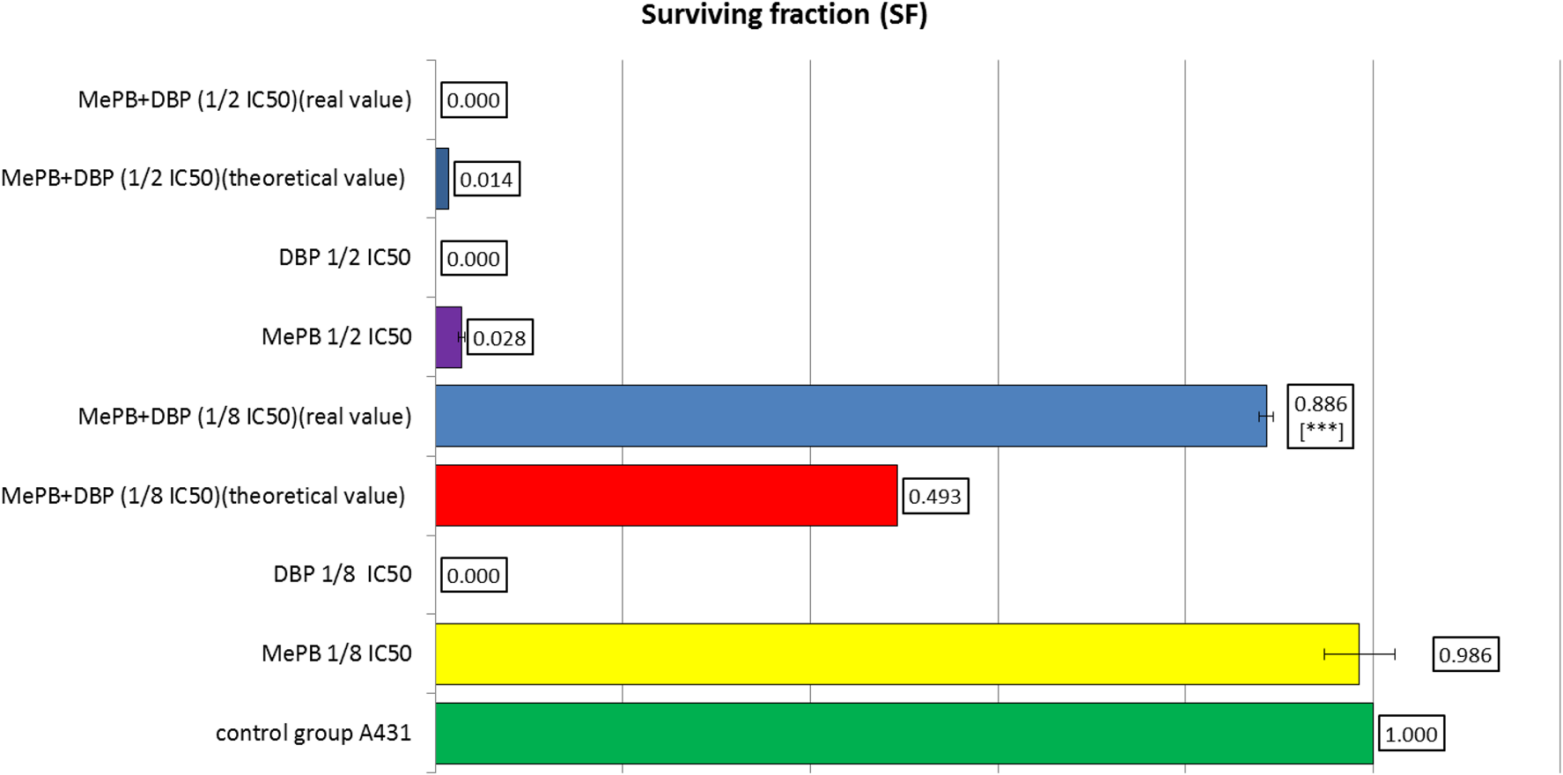
The effect of equitoxic mixtures of methyl paraben (MePB) and dibutyl phthalate (DBP) on the survival of cells capable of proliferation. Equitoxic mixtures of MePB and DBP at concentrations corresponding to 1/8 of the IC_50_ value of each of the compounds showed a higher SF than the theoretically calculated, while at higher concentrations the inverse relationship was observed. Data are expressed as number of colonies formed after exposure of cells to control (control = 1). Each bar represents the mean ± SD of three independent experiments, probability in one sample t-test or one sample Wilcoxon test: ***p < 0.001

The analysis of the CFEA test results showed that dibutyl phthalate applied individually to A431 cells, both at a concentration corresponding to 1/8 IC_50_ and at a concentration of 1/2 the value of the determined IC_50_ concentration, completely inhibited cell proliferation, and MePB was slightly less inhibitory at a concentration corresponding to 1/2 the IC_50_ value whereas at a concentration of 1/8 of its IC_50_, it almost did not inhibit proliferation (Fig. 3). Conversely, equitoxic mixtures of methyl paraben (MePB) and dibutyl phthalate (DBP) at concentrations corresponding to 1/8 of the IC_50_ value of each of the compounds showed a higher SF than the theoretically calculated, while at higher concentrations the inverse relationship was observed (Fig. 4).

### Holotomographic microscopy images

**Fig. 5:**
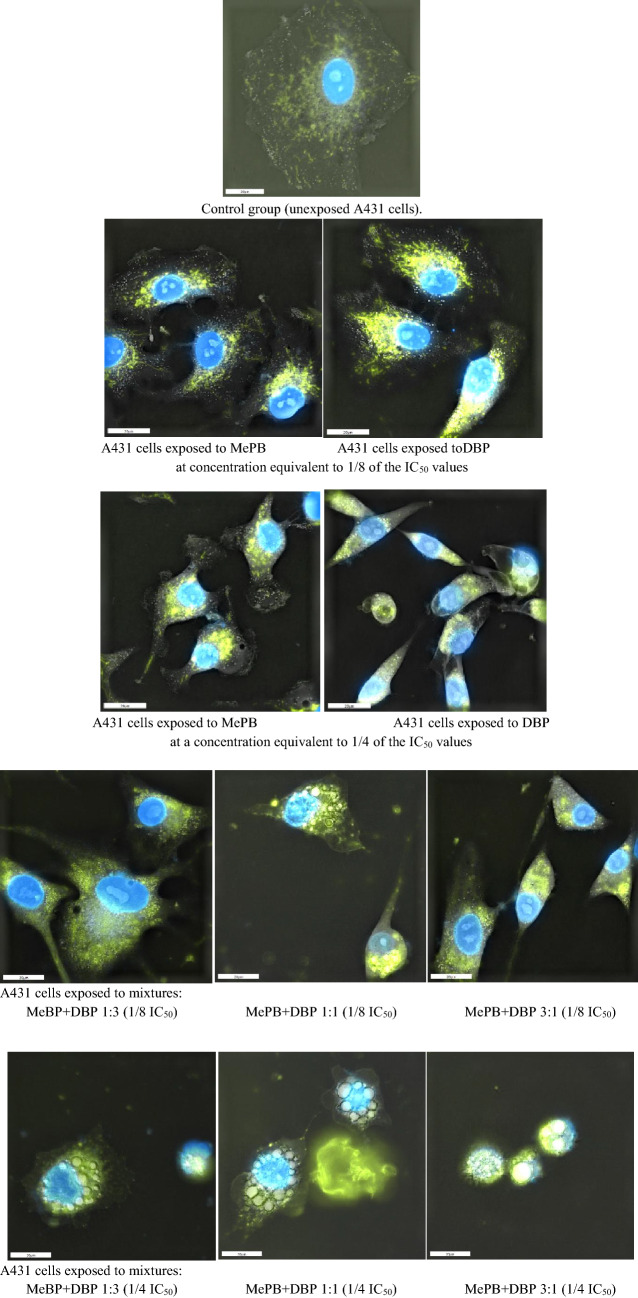
Holotomographic microscope images of A431 cells with three-dimensional images of their internal structure, individually exposed to dibutyl phthalate (DBP) and methyl paraben (MePB) at doses corresponding to 1/8 and 1/4 of their IC_50_ values, as well as to binary mixtures of these compounds in 1:3, 1:1 and 3:1 ratio. To visualise mitochondrial structures, the cells were incubated with MitoView (Biotium) dye at 100 nM, while nuclei were stained with DAPI reagent (1:1000, Sigma). The cells were incubated with dyes for 15 min at 37 °C. After staining the cell structures, observations of the cells were carried out under a holotomographic microscope. Pictures were taken under a magnification of 60x

Holotomographic microscopy images of A431 cells (not exposed) were compared to those exposed to dibutyl phthalate (DBP) and methyl paraben (MePB) to assess cellular changes due to exposure to reprotoxic/endocrine disrupting substances, and binary mixtures of these compounds at a ratio of 1:3; 1:1 and 3:1 in concentrations corresponding to 1/8 and 1/4 of the IC_50_ values were determined for these substances (Fig. 5). The HTM images show that healthy A431 cells (not exposed to the tested compounds/mixtures) are characterised by a regular shape, dense cytoplasm with worm-shaped mitochondria and regular intact cell nucleus membranes. After exposure of the A431 cells to the tested compounds (individually) at a concentration corresponding to 1/8 of the IC_50_ value, their morphological changes were observed in comparison with the control group (not exposed), which consisted of contracting the cytoplasm of cells, mitochondrial translocations and few changes in cell nuclei. Cells exposed to DBP and MePB in concentrations corresponding to 1/4 of the IC_50_ value were characterised by a highly contracted cytoplasm and mitochondria compacted within the cytoplasm and nucleus. The cells had an altered (elongated) shape compared to the controls. Mitochondrial swelling and dense granules in the matrix were also observed, as well as the onset of cytoplasmic vacuolisation. After the exposure to mixtures of dibutyl phthalate (DBP) and methyl paraben (MePB) at a concentration corresponding to 1/8 of the IC_50_ value, a progressive contraction of the cytoplasm of cells was observed. Exposing the cells to a higher concentration of the tested mixtures (1/4 IC_50_) caused the destruction of the organelles inside the cells and changes in the shape of the cell nucleus, which in turn, led to cell necrosis. When the cells were exposed to higher concentrations of DBP and MePB applied individually to the cells, the changes appeared slightly less pronounced than when the cells were exposed to binary mixtures of these compounds in all ratios tested.

### Results of apoptosis markers evaluation

In consideration of the above observations, the evaluation of caspase-3/7 activity in A431 cells after exposure to dibutyl phthalate (DBP) and methyl paraben (MePB) was performed using the IncuCyte^®^ S3 Live-Cell Analysis System individually and in two-component equitoxic mixtures during 24 h real-time observation.

**Fig. 6:**
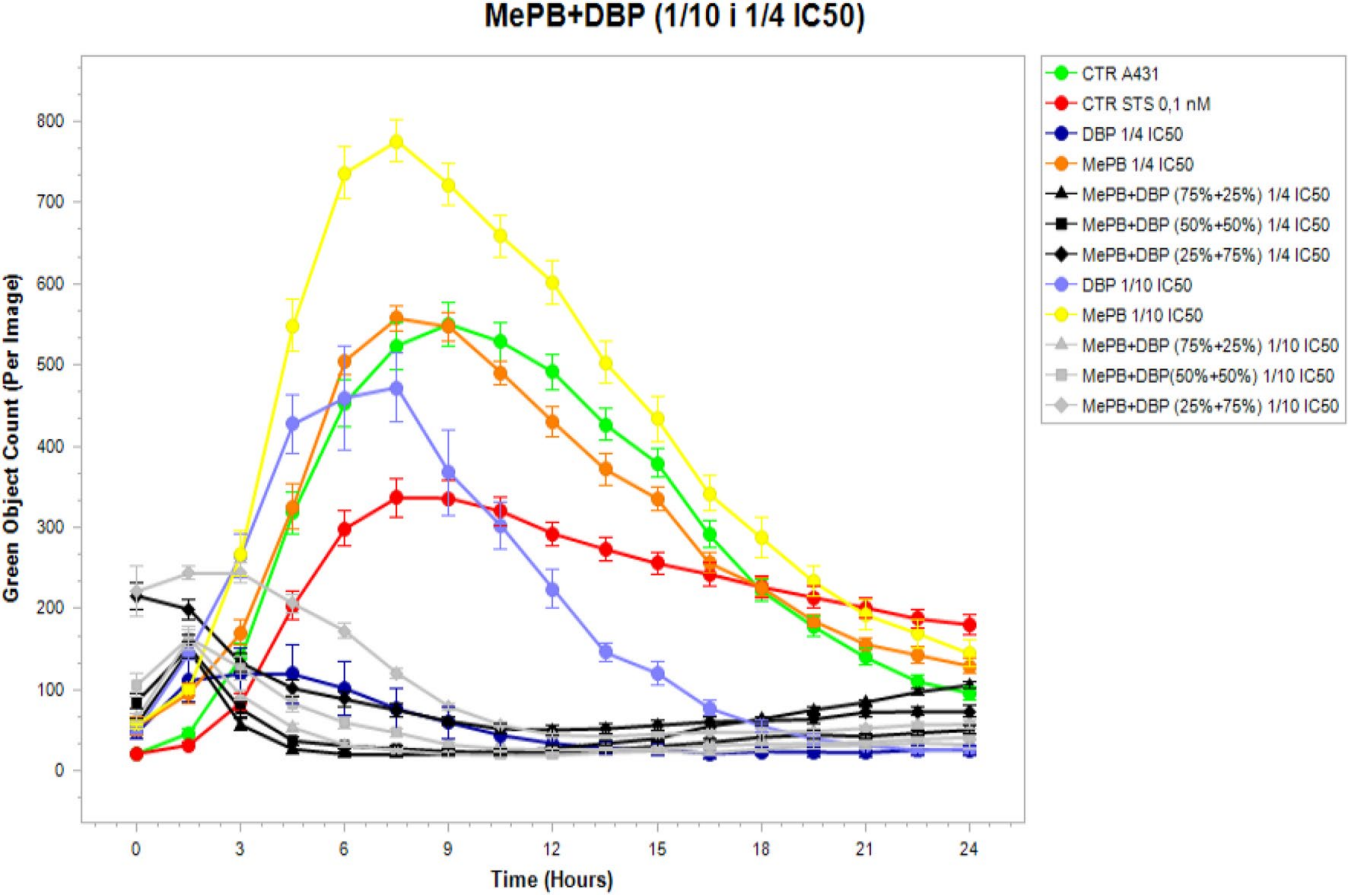
Activity of caspase-3/7 over time after exposure of A431 cells to dibutyl phthalate (DBP) and methyl paraben (MePB) at concentrations corresponding to 1/4 and 1/10 IC_50_ values, and their binary equitoxic mixtures. Evaluation was performed using the IncuCyte^®^ S3 Live-Cell Analysis System during 24 h real-time observation. Cells treated with staurosporine (0.1 nM, Sigma-Aldrich) were used as a positive control. The images were analysed for the number of green objects (fluorescing cells) per well by the algorithm in the IncuCyte S3 Software (v2018B). Each experiment was performed in three independent replications

During the observation of the A431 cells, it was found that the highest caspase activity was characterised by those cells that were exposed to methylparaben (MePB) at the lower concentration tested. This activity increased starting at the time the cells were exposed to the compound and reached a maximum value after 6–8 h. It was also observed that the A431 cells as such (not exposed) were characterised by high enzymatic activity of caspases (it exceeded the activity of caspases in cells exposed to a model pro-apoptotic compound—staurosporin). It was also found that during the first day after exposure, the caspase activity in the A431 cells exposed to mixtures of MePB and DBP in all proportions was significantly lower than in these cells exposed to individual compounds at concentrations corresponding to 1/10 of their IC_50_ values. At the higher of the tested concentrations (1/4 IC_50_), low caspase activity was observed both in cells exposed to the test mixtures in all proportions and in cells exposed to DBP alone (Fig. 6).

**Fig. 7:**
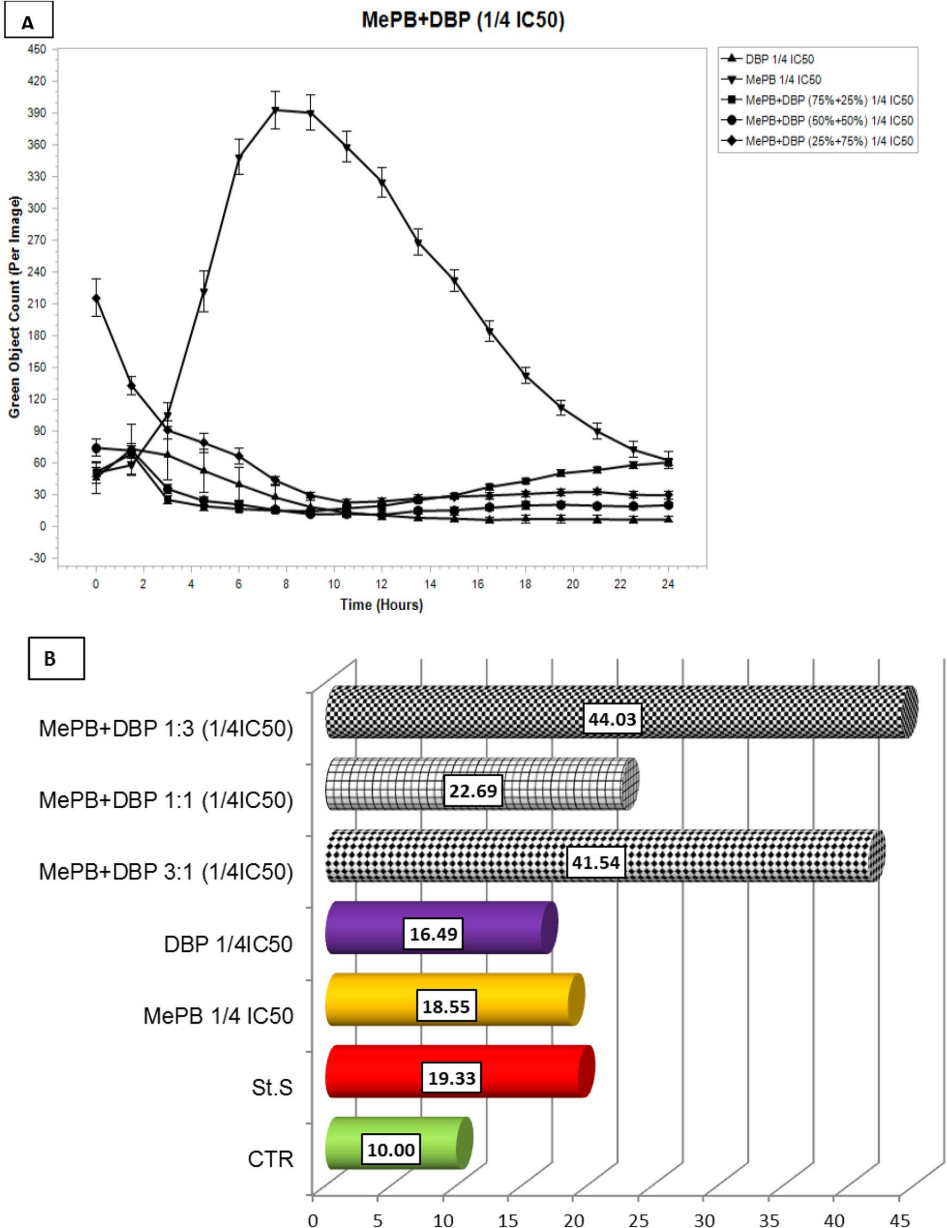
Analysis of caspase-3/7 activity in A431 cells exposed to MePB, DBP and their mixtures in concentrations equivalent to ¼ IC_50_ values (**a**) using a real time analysis system (IncuCyte S3) during 24 h real-time observation. The images were analysed for the number of green objects (fluorescing cells) per well by the algorithm in the IncuCyte S3 Software (v2018B). Each experiment was performed in three independent replications (**b**) based on an apoptosis rate counted after 24 h exposure using a Nucleocounter NC-3000 by the FLICA method. The assay involved the carboxyfluorescein-labelled (FAM) four-amino acid (Asp-Glu-Val-Asp) substrate conjugated with a fluoromethyl ketone residue (FMK), which determined its penetration through the cell membrane into the cell interior and irreversible binding of the specific amino acid sequence to the active centre of the caspase. Any unbound inhibitor was washed away and as a result labelled, and only cells in which the caspase has been activated were detected using a fluorescence reader

**Fig. 8:**
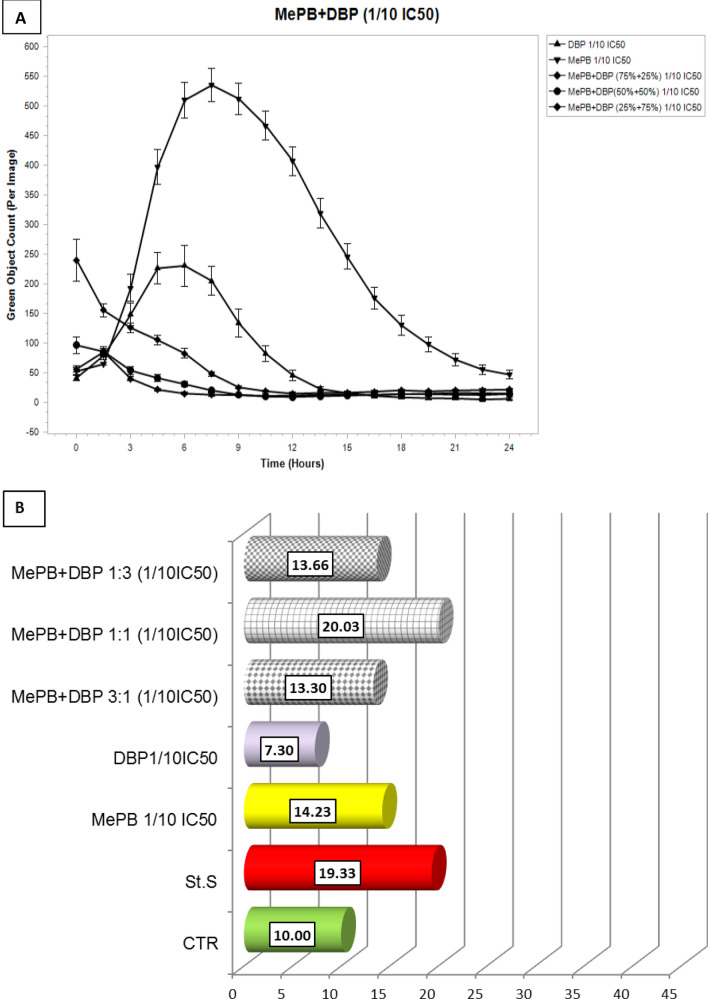
Analysis of caspase-3/7 activity in A431 cells exposed to MePB, DBP and their mixtures in concentrations equivalent to 1/10 IC_50_ values (**a**) using a real time analysis system (IncuCyte S3) during 24 h real-time observation. The images were analysed for the number of green objects (fluorescing cells) per well by the algorithm in the IncuCyte S3 Software (v2018B) during 24 h real-time observation. The images were analysed for the number of green objects (fluorescing cells) per well by the algorithm in the IncuCyte S3 Software (v2018B). Each experiment was performed in three independent replications (**b**) based on an apoptosis rate counted after 24 h exposure using a Nucleocounter NC-3000 by the FLICA method. The assay involved the carboxyfluorescein-labelled (FAM) four-amino acid (Asp-Glu-Val-Asp) substrate conjugated with a fluoromethyl ketone residue (FMK), which determined its penetration through the cell membrane into the cell interior and irreversible binding of the specific amino acid sequence to the active centre of the caspase. Any unbound inhibitor was washed away and as a result labelled, and only cells in which the caspase has been activated were detected using a fluorescence reader

By comparing the real-time (24-h) assessment of caspase-3/7 activity using a IncuCyte^®^ S3 Live-Cell Analysis System with an apoptotic rate calculated from the caspase-3/7 activity, determined with a NC 3000, after 24 h of exposure (Figs. 7, 8), it was found that the maximum activity of caspase-3/7 in A431 cells is observed 6–8 h after exposure to MePB at both tested concentrations and DBP at a concentration corresponding to 1/10 of the IC_50_ value, followed by a decrease in their activity. Therefore, the rates of apoptosis calculated after 24 h of exposure (b) do not reflect the peak enzyme activity. To estimate the effect of the tested compounds individually and in mixtures, it seems advisable to assess the level of this apoptosis marker after 6–8 h of exposure.

The results of 24/7 real-time caspase activity assay using a IncuCyte^®^ S3 Live-Cell Analysis System in A431 cells exposed to MePB and DBP singly and in binary mixtures (1:3; 1:1 and 3:1) may suggest an anti-apoptotic effect (assessed on caspase-3/7 activity) of co-exposure to the lower concentrations of the reproductive/endocrine disrupting compounds tested.

**Fig. 9:**
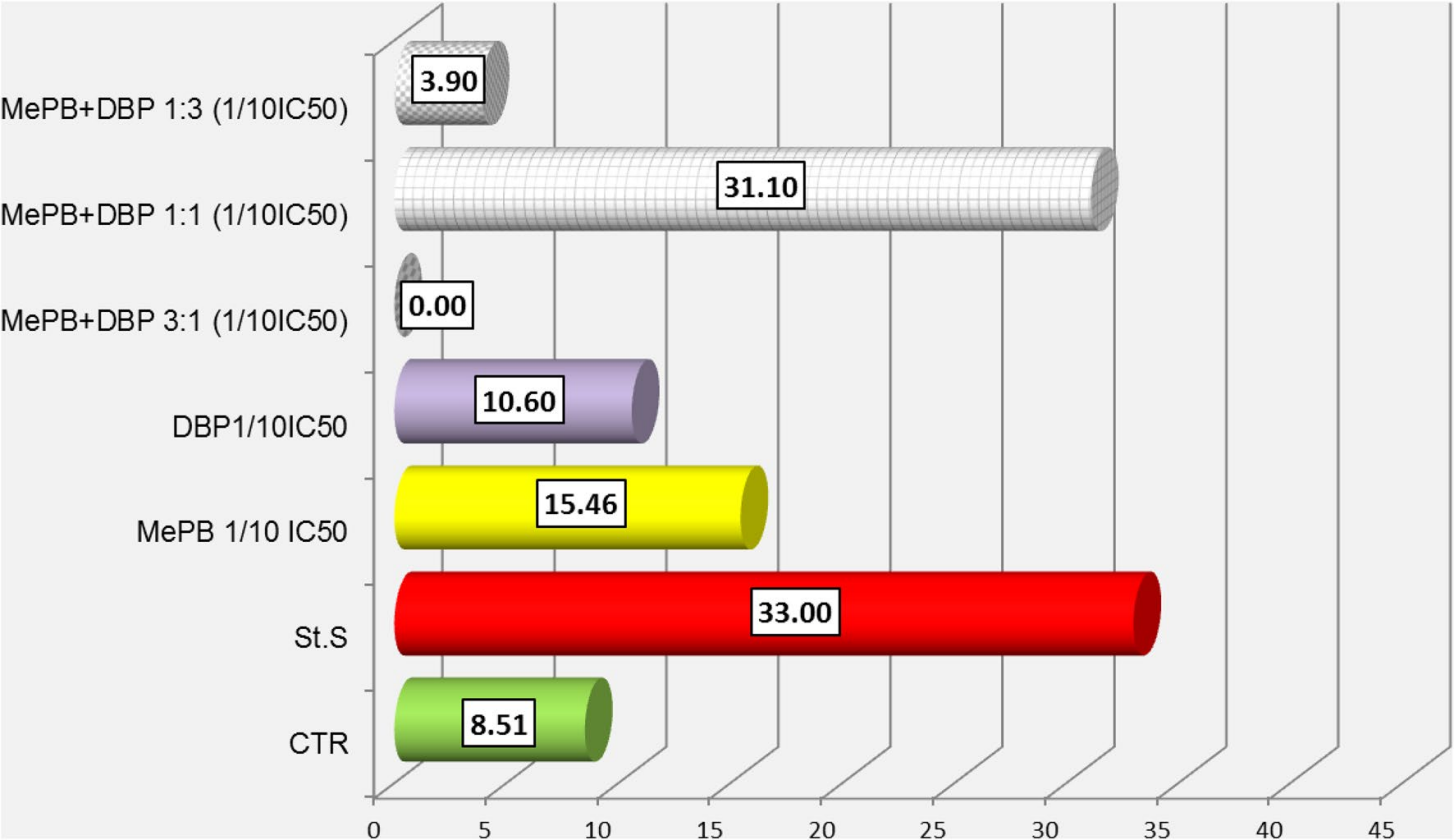
Apoptosis rate based on Annexin V binding in A431 cells 24 h after exposure to MePB and DBP individually and in 1:3 mixtures; 1:1 and 3:1 at concentrations corresponding to 1/10 IC_50._ The translocation of phosphatidylserine in cells was assessed using Nucleocounter NC 3000 on the basis of binding the fluorescently labelled Annexin V conjugated with fluorescein-FITC isotiocyanate

**Fig. 10:**
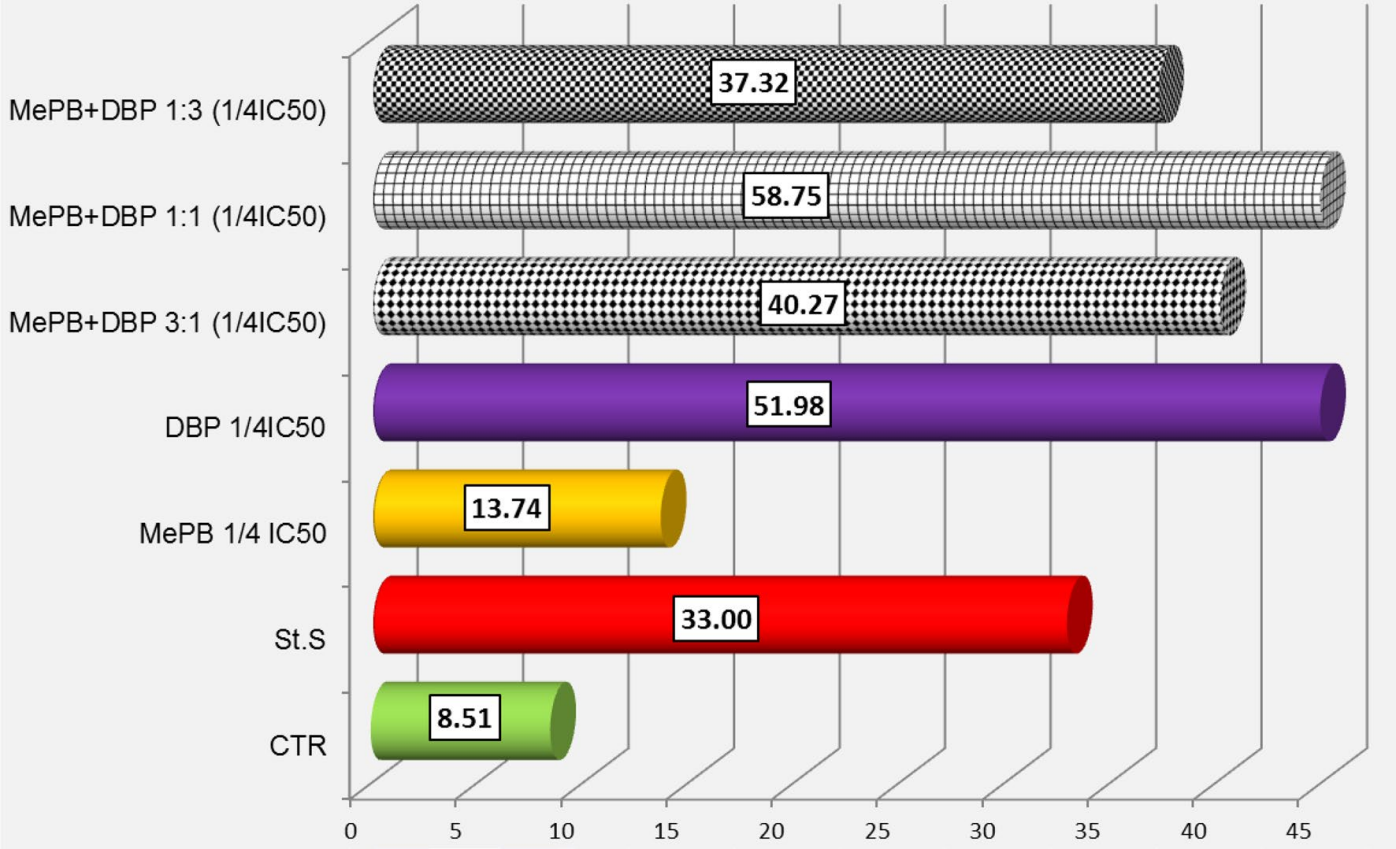
Apoptosis rate based on Annexin V binding in A431 cells 24 h after exposure to MePB and DBP individually and in 1:3 mixtures; 1:1 and 3:1 at concentrations corresponding to 1/4 IC_50_. The translocation of phosphatidylserine in cells was assessed using Nucleocounter NC 3000 on the basis of binding the fluorescently labelled Annexin V conjugated with fluorescein-FITC isotiocyanate

The values of the apoptosis rate determined on the basis of Annexin V binding in A431 cells after exposure to MePB and DBP separately and in mixtures at a concentration corresponding to 1/10 of their IC_50_ values were very low for cells exposed to the mixtures in molar ratios 1:3 and 3:1, while the apoptosis rate has reached a value comparable to the value obtained for the positive control (staurosporine) for cells exposed to the equimolar mixture (1:1) (Fig. 9). The high apoptosis rates calculated for all three mixtures of tested compounds at concentrations corresponding to 1/4 of their IC_50_ values were similar to the value obtained for DBP applied to cells alone, which could suggest that rather dibutyl phthalate than methyl paraben could be responsible for the membrane conformational changes and apoptosis in the tested mixtures (Fig. 10).

## Discussion

The results of research on possible interactions between reprotoxic substances/endocrine disruptors available in the literature indicate an ambiguous direction of changes in their action in mixtures. Therefore, for example, the additive effect of a mixture of endocrine disrupting phenolic compounds: 4-tert-octylphenol, bisphenol A, 4-nonylphenol and 2,4-dichlorophenol was found in fish by *Carassius auratus* Li et al. [30]. Couleau et al. [31] found that exposure of differentiated cells of the monocytic-macrophage line THP-1 to the bisphenol A (BPA) or dibutyl phthalate (DBP) induced a reduction in the severity of phagocytosis (for BPA in a concentration-dependent manner), while co-exposure to BPA and DBP also induced a concentration-dependent inhibition of this parameter, but without any interactions (compared to BPA or DBP alone). In contrast to exposure to diethylhexyl phthalate (DEHP) or 4-tert-octylphenol (4-OP) alone and the combination of BPA + DEHP, which altered the ability to phagocytose, but not in a concentration-dependent manner, simultaneous exposure to DEHP and 4-OP induced a concentration-dependent reduction in phagocytic capacity, with an additive effect only observed for this mixture at the highest concentration. Greater than for each substance separately, the severity of the adverse effect of bisphenol A impairing progesterone synthesis was also observed in its mixture with the flavonoid fisetin in the studies by Bujnakova Mlynarcikova and Scsukova [32]. In turn, in the studies of the combined effects of butyl paraben and triclosan and their toxicity to the gonads of male Wistar rats, conducted by Riad et al. [33], it was found that combined exposure to the tested compounds revealed reproductive toxicity that was less than additive. Butyl paraben and triclosan administered alone caused a significant increase in the level of estradiol in the blood, while the application of the compounds in the mixture did not change its level. The activity of superoxide dismutase was significantly reduced in the group of animals treated with butyl paraben, increased in the group exposed to triclosan, while it remained only statistically insignificant in the group receiving the paraben-triclosan mixture. However, antagonism of action was found between triclosan and triclocarban [34], which indicates the possibility of the occurrence of interactions between the components of cosmetics and /or chemical industry products that are difficult to predict (without empirical studies), significantly modifying not only the image of the total exposure to non in vitro cells, but also the effects on the organism of humans exposed to their mixture: an employee of the cosmetic or chemical industry, as well as the user of the product which contains the mixture.

It should be noted that researchers dealing with chemical interactions make it clear that the risk assessment of chemical compounds based on single substance data can lead to an underestimation of the risk, and therefore the effects should always be estimated on the basis of combined exposure studies. Considering that these EDCs are widely used in industry, human exposure to these chemicals is ubiquitous—organisms are constantly exposed to complex mixtures of EDCs that simultaneously affect various systems and organs (not just the endocrine system), making it difficult to study disturbances caused together by individual factors. An additional argument that may strengthen the justification of the need to study the combined effects of substances that may appear simultaneously in the work environment are the results of the research of Christen et al. [35] on the MDA-kb2 human breast cancer cell line, who observed various interactions in binary mixtures of phthalates and bisphenol A, which depended on concentrations of the tested compounds: the synergism of action in terms of antiandrogenic activity occurred at high concentrations of the tested compounds in the mixture, while the compounds contained in the binary mixtures at low concentrations showed a tendency to antagonist activity. Synergy effect of toxic substances could be especially crucial for the risk assessment and principles established for calculating the combined exposure effect using the total exposure factor (calculated as the sum of the quotients of the concentrations of individual substances and the corresponding OEL values) which do not apply to substances having a synergistic effect, because it does not adequately represent the risk involved (real risk is higher than calculated in this manner). The underestimation of risk in combined exposure to chemicals has been discussed in the literature to this day [36]. There is a need in documenting real working life situations of multiple and coexposures in line with the rising attention given to ‘exposome’ and its potential relevance for reflecting workplace exposures [37]. This concept also raises other concerns, including potential for confounding and identifying synergistic or additive associations between multiple exposures and occupational health.

The results of the research carried out in this study confirm that the final result of the action of the ‘cocktail’ of chemicals harmful to reproduction/endocrine system may be significantly changed by the interactions. Modification of actions may concern not only strength and time, but also the direction of the toxic action of such xenobiotics. The synergistic interactions between the tested substances observed in this study are a phenomenon frequently encountered in the case of joint exposure to chemical substances. A synergistic effect, characterised by the fact that the simultaneous action of two compounds is greater than the sum of the effects of each of these substances separately, was found in terms of cytotoxicity by assessing the metabolic activity of cells and the integrity of the cell membrane for the tested equimolar mixture (1:1).

Our synergism observation is consistent with the results of the studies by Zhang et al. [38], who observed the synergistic effect of a mixture of BPA and DBP, consisting in increasing expression of the androgen receptor (AR), the progesterone hormone receptor (PR) and the gonadotropin-releasing hormone receptor (GNRHR) in rats (genes of the hypothalamus). These observations are also consistent with the statement that DBP induction of apoptosis and neurotoxicity is mediated by the aromatic hydrocarbon receptor (AhR; aryl hydrocarbon receptor) (it is activated by environmental pollutants), and not mediated by ERα (Oestrogen Receptor Alpha) or ERβ (Oestrogen Receptor Beta) or PPARγ (Peroxisome Proliferator-Activated Gamma Receptor) [39], but there is a correlation between the metabolic pathways activated by the attachment of a xenobiotic to the AhR receptor, or 17β-oestradiol (E2) to the ER receptor, also called ‘receptor talk’ or transactivation, which can completely change the signal transduction pathway, leading to unpredictable consequences [40]. This appears important considering the fact that parabens also exert an oestrogenic effect. On the basis of the research of Engeli et al. [41], it was found that parabens interfere with 17beta-hydroxysteroid dehydrogenases (17β-HSD: oestrogen-activating enzyme 17*β-*hydroxysteroid dehydrogenase), which modulate the biological strength of oestrogens and androgens by interconversion of inactive 17-keto-steroids and their active 17-beta-hydroxy equivalents (the inhibitory effect on 17β-HSD1 is directly proportional to the size of the paraben). Harris et al. [42] found that most phthalates exhibit extremely weak oestrogenic activity. The relative oestrogenic potency of these compounds decreases in the following order: butyl benzyl phthalate (BBP) > dibutyl phthalate (DBP) > diisobutyl phthalate (DIBP) > diethyl phthalate (DEP) > diisononyl phthalate (DINP). These authors did not observe synergy of action in mixtures of BBP, DBP and 17beta-estradiol in the screening of recombinant strains of Saccharomyces cerevisiae, although the effects of the mixtures were approximately additive. Studies by Evans et al. [43] revealed that chemicals that exhibit minimal oestrogenicity can reduce (negatively modulate) the effect of an oestrogen mixture. Whether the observed type of modulation will be effective, according to the authors, it most likely depends on the concentrations of chemical substances used, and requires information on the likely concentrations of oestrogens in human tissues and on potential modulators.

A surprisingly frequent in practice phenomenon of change in the direction of interactions between chemicals seems particularly interesting. This type of observations were made, among others, by Christen et al. [35] investigating the antiandrogenic activity of six binary and one ternary mixture of phthalates and binary mixtures of phthalates and bisphenol A in MDA-kb2 cells, a line derived from a breast cancer cell line. They observed that the activity of the mixtures tended to be synergistic at high concentrations and antagonistic at low concentrations of the ingredients of the mixtures. The isobole images confirmed the synergistic effect of mixtures of benzyl butyl and dibutyl phthalates (BBP + DBP); dibutyl and diethyl phthalates (DBP + DEP) and diethyl phthalate and bisphenol A (DEP + BPA) in high concentrations, while the antagonism between the components of BBP + DBP, BBP + DEP, DBP + DEP and DBP + BPA mixtures in low concentrations.

In our study, a phenomenon was observed in which the synergistic effect of co-exposure of A431 cells to methyl paraben and dibutyl phthalate in terms of cytotoxicity assessed using an MTT test changed into antagonism with increasing of DBP concentration in the mixtures. Antagonistic activity was also observed in the evaluation of the long-term consequences of exposure of A431 cells and their proliferation capacity in a clonogenic assay for mixtures containing dibutyl phthalate (DBP) at the lower concentration tested.

Taking into consideration Li et al. [44] in vitro studies on Sertoli cells, analysing the combined effect of nonylphenol (NP) and di-n-butyl phthalate (DBP), which found additivity of the cytotoxic effect of the tested compounds in the mixture, as well as induction of the apoptosis process by the mixture which the DBP itself did not cause, our study also involved apoptosis markers in cells exposed to single substances and their mixtures. The results of the 24/7 real-time caspase activity assay using an IncuCyte^®^ S3 Live-Cell Analysis System in A431 cells exposed to MePB and DBP singly and in binary mixtures (1:3; 1:1 and 3:1) may suggest an anti-apoptotic effect (assessed on caspase-3/7 activity) of co-exposure to the lower concentrations of the reproductive/endocrine disrupting compounds tested. It can also be concluded that to estimate the effect of the tested compounds individually and in mixtures, it seems advisable to assess the level of the apoptosis marker after 6–8 h of exposure.

In summary, it is worth noting that hypotheses are being formulated increasingly more often [45] that exposure to mixtures of endocrine disrupting compounds causes either synergistic or antagonistic effects, which, however, in both cases may cause undesirable effects that are not reflected in single compound exposure.

It must be emphasized that in this work we used simple method based on synergy indexes and isobolographic analysis after IC_50_ determination and the prediction of combined toxicity used in this research can be different with practical data, hence the advanced calculation of toxicity prediction could be necessary (e.g. mathematical approach and modern statistical models as in Yang et al. [46] or D'Almeida et al. [47] research). The exposure assessment (the first step of risk assessment) and identification of hazards at the workplace at the presence of mixtures requires a careful study of the workplace and physical and toxicological principles to ensure a comprehensive evaluation [48]. It is important to acknowledge this when considering the effects of combined exposure in risk assessment in order to not-underestimate the risk of adverse effects associated with exposure to chemical mixtures [49]. This study contributes to the body of part of knowledge on the toxicology of mixtures and gives some preliminary data and one more evidence of the paramount importance on mixture toxicity studies. Further research on the action of a combined 'cocktail of chemicals' and the mechanisms responsible for interactions is needed.
